# Visual outcome of mega-dose intravenous corticosteroid treatment in non-arteritic anterior ischemic optic neuropathy – retrospective analysis

**DOI:** 10.1186/1471-2415-14-62

**Published:** 2014-05-03

**Authors:** Michael Kinori, Iris Ben-Bassat, Yael Wasserzug, Angela Chetrit, Ruth Huna-Baron

**Affiliations:** 1 Neuro-Ophthalmology unit, The Goldschleger Eye Institute, Sheba Medical Center, Tel- Hashomer, Israel affiliated to the Sackler school of medicine, Tel Aviv university, Tel Aviv, Israel; 2 The Pinchas Borenstein Talpiot Medical Leadership Program, Sheba Medical Center, Tel Hashomer, Ramat Gan, Israel; 3 Gertner Institute for Research and Epidemiology, Sheba Medical Center, Tel Hashomer, Ramat Gan Israel

**Keywords:** Anterior ischemic optic neuropathy, Corticosteroids, Visual acuity, Visual field, Mean deviation

## Abstract

**Background:**

To date, non arteritic anterior ischemic optic neuropathy (NAION) is still incurable. We wish to evaluate the effect of intravenous (IV) corticosteroids on the visual outcome of NAION patients.

**Methods:**

Visual parameters were retrospectively compared between NAION patients treated with IV corticosteroids and untreated NAION patients (control). Visual acuity (VA) and Humphrey automated static perimetry visual field (VF) defects of the affected eye were compared between groups at baseline, 1, 3, 6 months, and end of follow-up visits. The VF analysis consisted of number of quadrant involvements and mean deviation (MD).

**Results:**

Each group comprised 23 patients (24 eyes). Mean initial VA was similar in the control and treatment groups (p = 0.8). VA at end of follow-up did not improve in either groups (p = 0.8 treated group, p = 0.1 control group). No improvement and no difference in VF defects were found by either quadrant analysis (p = 0.1 treated group, p = 0.5 control group) or MD analysis (p = 0.2, treated group, p = 0.9 control group). VA and VF parameters tended to be worse in the treated group, although without statistical significance.

**Conclusions:**

Our results suggest that IV corticosteroids may not improve the visual outcome of NAION patients. Since intravenous corticosteroids could potentially cause serious adverse effects, this treatment for NAION is questionable.

## Background

Nonarteritic ischemic optic neuropathy (NAION) is the most common cause for acute optic neuropathy in adults over the age of 50 [1]. It is believed to be the result of ischemic damage to the anterior optic nerve that is predominantly supplied by the posterior ciliary arteries [2,3]. Most patients present with acute unilateral painless visual defect involving mainly, but not only, the inferior visual field. To date, there is no generally accepted, well-proven, effective treatment for this condition. The only randomized control study for the treatment of NAION was the IONDT (Ischemic Optic Neuropathy Decompression Trial) [4] which suggested that optic nerve decompression surgery for NAION patients is ineffective, and may even be harmful. Other studies explored the role of aspirin [5-7], vasodilators [8], heparin-induced extracorporeal LDL/fibrinogen precipitation (HELP) [9], hyperbaric oxygen [10], diphenylhydantoin [11], norepinephrine [12], levodopa [13], topical brimonidine [14,15], intravitreal bevacizumab [16,17] and systemic corticosteroids [18-20]. Recently Prokosch et al. showed that adding the corticosteroid floucortolone to their standard treatment (intravenous and per os pentoxifylline for one week and then per os for a further 6 months) slightly improves the short and long term visual acuity (VA) in some patients. However, visual field (VF) was not improved in either group [20].

The rationale behind corticosteroid treatment, although not proven, is the thought that faster resolution of optic disc edema may be associated with better visual outcome [21]. The presumed mechanism for corticosteroids improve the outcome in NAION patients is prevention of the “vicious circle” [19] in which the ischemic tissue further suffers from the secondary damage by a mechanical pressure caused by the swollen ischemic axons in an already crowded disc with a small scleral canal. This would not prevent the primary insult but should theoretically limit the secondary insult. Reducing capillary permeability in the optic disc by corticosteroids [21] could be another mechanism.

Recently, Hayreh reported a very large study carried out over a period of 27 years [19]. This study comprised NAION patients who were treated with systemic oral corticosteroids as opposed to untreated NAION patients. Although the reported results favored treatment with 80 milligram prednisone for 2 weeks with subsequent tapering, with regard to VA and VF performances, it is still not widely accepted. In fact, a thought-provoking discussion in the literature was recently conducted on this issue [19,22,23].

No controlled studies of megadose intravenous (IV) corticosteroids (1 gr/day methylprednisolone) for NAION have been performed. Some clinicians tend to recommend this approach in severe progressive cases in order to decrease the secondary neural damage, despite no supporting evidence in the literature [3].

This study was conducted to explore the visual outcome in NAION patients treated with IV corticosteroids as compared to untreated patients, and to report the adverse effects of such treatment.

## Methods

The study was approved by the local Ethics Committee of The Chaim Sheba Medical Center, Tel Hashomer, Israel. We conducted a retrospective chart review of all patients diagnosed as NAION according to the IONDT criteria: Sudden loss of vision within the previous 14 days, a relative afferent pupillary defect, optic disc edema and an abnormal VF consistent with optic neuropathy. The only exception was the VA parameter in the IONDT study (20/64 or less in the affected eye) which was not applied in our study. The arteritic type of ischemic optic neuropathy (A-AION) was ruled out in all patients using clinical and laboratory data, mainly erythrocyte sedimentation rate, C-reactive protein and blood count. Inclusion criteria were: 1) Diagnosis of NAION according to IONDT [4] 2) Rapidly progressive NAION; or 3) Poor vision in the contralateral eye. Exclusion criteria were: 1) Previously documented retinal conditions that could influence VA, such as severe nonproliferative, or proliferative diabetic retinopathy. Patients with mild nonproliferative diabetic retinopathy were included; 2) Glaucoma patients with documented previous VF defects; 3) Patients with follow-up period of less than 6 months, and 4) Patients with unreliable VFs.

All patients were examined at the Neuroophthalmology Clinic in the Chaim Sheba Medical Center, Israel. Patients with progressive NAION, or poor vision in the contralateral eye without a major systemic condition, such as uncontrolled hypertension, uncontrolled diabetes, or congestive heart failure were offered treatment with IV corticosteroids protocol as performed in the optic neuritis treatment trial (ONTT) [24]. All patients in the intervention group had to sign an informed consent before treatment, which was given within 2 weeks of onset. The control group included patients with NAION who refused the treatment or those who had systemic contraindications for corticosteroid treatment. We made efforts to include in the control group patients with similar characteristics as the treated group (e.g. age, gender, number of cardiovascular risk factors, aspirin use and crowded disc).

Data for analysis included examinations conducted at baseline, 1, 3, 6 months, and end of follow-up. Visual parameters were assessed by VA using the standard Snellen acuity chart (converted to LogMAR for statistical analysis), and Humphrey automated static perimetry for VF defects. The latter were graded by two methods: a) a scale of 0 to 4 (0 being a normal field and 4 being a defect involving four quadrants); b) mean deviation (MD).

Statistical analysis of the data was performed by Student’s t test for continuous variables (such as VA and MD) and Fisher’s exact test for categorical variables (such as number of quadrant involvement in VF) ± values represent standard deviation.

## Results

**Figure 1:**
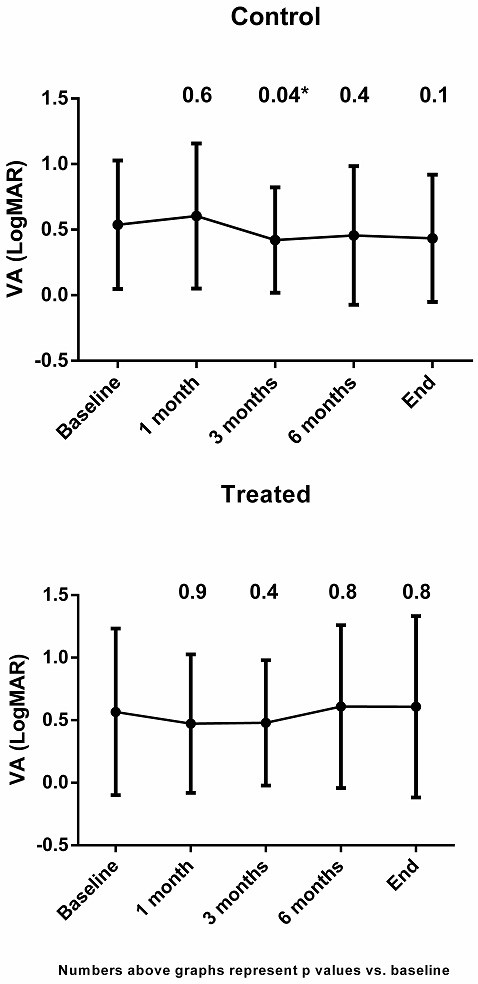
**Visual acuity (VA) in LogMAR units in the control group (upper) and IV methylprednisolone treated group.** Value above lines represents p-value of mean LogMAR VA compared to mean baseline VA at different time points. Baseline mean VA was the same in both groups: Log MAR 0.54 (equals to 20/70). The final VA was not statistically different form baseline in both groups: 0.43 (equals to 20/53) in the control group (p = 0.1 compared to baseline) and 0.61 (equals to 20/80) in the treated group (p = 0.8 compared to baseline). Note a very mild trend toward VA improvement in the control group versus worsening in the treated group, although statistically not significant.

Main demographic and clinical data of the study population is depicted in Table 1. Groups were similar regarding age, gender, contralateral optic disc appearance and the presence of vascular risk factors. Follow-up was slightly longer for the control group (mean, 36 months versus 22 months in the treatment group). Mean initial VA was 20/70 (LogMAR 0.54 ± 0.67) in the treated group and 20/69 (LogMAR 0.54 ± 0.49) in the control group (p = 0.8). Results of VA performances over time for both groups are shown in Figure 1. In the control group VA remained the same at all-time points other than at 3 months after baseline where a slight improvement was seen. However, this improvement was probably clinically insignificant (average improvement from 20/66 to 20/53, p = 0.04). Moreover, after Bonferroni correction for multiple comparisons this results turned out to be insignificant (p = 0.12). In the IV treated group the average VA also remained the same, but with a trend towards an exacerbated final VA compared to the control group. The average VA at end of follow-up was 20/80 (0.60 in LogMAR) in the treated group and 20/53 (0.42 in LogMAR) in the control group (p = 0.3).

**Table 1:** Main demographic and clinical data of the treated and control group

	**Treated group**	**Control group**	**P value**
**Male: female**	14:10	16:8	0.55
**Age (years)**	54.4 ± 12.3	55.4 ± 9.6	0.78
**Follow-up (months)**	22.7 ± 23.4	36.2 ± 24.2	0.04
Number vascular risk factors*	1.4 ± 1.1	1.6 ± 1.1	0.60
**Crowded disc**	10 (42%)	13 (54%)	0.31
**Mean VA (LogMAR)**	0.54 ± 0.67	0.54 ± 0.49	0.80
**Visual field parameters**			
**Mean quadrant involvement**	2.4 ± 0.8	2.0 ± 0.6	0.007
**Mean MD**	9.7 ± 10.4	9.3 ± 10.5	0.9

VA = Visual acuity, MD = Mean deviation.

*Includes: Hypertention, diabetes mellitus, ischemic heart disease, dyslipidemia and smoking.

**Figure 2:**
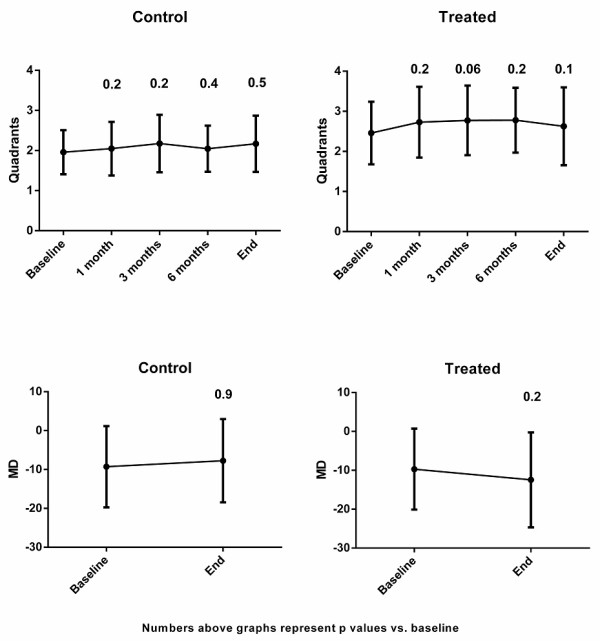
**Humphrey visual field (HVF) analysis in quadrants (upper graphs) and according to the mean deviation (MD) (lower graphs).** Value above columns represents the p-value of mean quadrant involvement (upper graphs) and mean MD value (lower graphs) compared to baseline. Note that in both groups there was no change in the number of quadrant involvement at any time point. Analysis by MD showed no statistically significant difference in both groups between baseline and final values (p = 0.9 for controls, p = 0.2 for treated group). Note a mild trend toward improvement in the control group versus worsening in the treated group in the MD analysis. This trend was not shown in the analysis by quadrants involvement. *After bonferroni correction for multiple comparisons: p = 0.12.

Figure 2 shows the outcome for VF according to quadrants involved and the average MD. The initial VF showed defects in 2.4 ± 0.8 in the treated group and 2.0 ± 0.6 quadrants in the control group. This difference was statistically significant (p = 0.007), and could be explained by the bias of the clinician to treat the more severe cases with IV corticosteroids. As shown, VF defect severity remained the same in both groups throughout the follow-up period. At final visit, quadrant involvement was 2.6 ± 0.9 in the treated group and 2.2 ± 0.7 in the control group (p = 0.07). In both groups mean VF quadrant involvement was not statistically significant from baseline in all examinations. The VF defects according to MD analysis showed no statistically significant difference in the initial MD value between groups (p = 0.9), as opposed to analysis by quadrants. No statistical difference was found between groups at the end of follow-up (p = 0.2).

Drug-related side effects in the treated group were minimal and observed in four patients: two patients suffered from a temporary increase in their blood glucose levels, one had myalgia, and one suffered from stomachaches.

## Discussion

Lack of available treatment for patients with NAION is a source of discomfort for the neuroophthalmologist. Various agents and procedures for NAION treatment have been suggested, but most without encouraging results.

The idea of treating NAION with corticosteroids is that relieving the pressure on the axons during the acute phase (when the optic disc is edematous) may prevent further damage to the optic nerve. Therefore, in most studies corticosteroids were administered in the acute phase, which is believed to be within the first 2 weeks [19]. This therapeutic window is also supported by animal models [25] as well as the common clinical experience of general progression in visual loss during this period, with stabilization thereafter [3]. For this reason the IONDT [4] also allowed a 2-week therapeutic window for the decompression to be made (for regular-entry patients). In our study all patients also received treatment within 2 weeks of onset.

The largest series to date reporting corticosteroid treatment for NAION [19], was conducted over a period of 27 years. This study included 613 NAION patients who were almost equally divided into two groups, oral corticosteroid treatment versus no treatment. Results showed that treatment was more beneficial: VA improved in 70% of the treated group as compared to 40% of the untreated group. Moreover, VF improved in 40% in the treated group versus 25% in the untreated group. The results were similar after 6 months and 1 year, which led the authors to conclude that treating NAION patients in the acute phase of the disease, and the sooner the better, is of major benefit, especially for patients with lower baseline performances. Admirable, we suspected that our clinical experience with NAION patients treated with systemic corticosteroids is less encouraging, similar to the report of Rebolleda et al. [26].

The aim of our study was to evaluate whether IV corticosteroids are beneficial for NAION patients. It is feasible that if the optic nerve could be “saved” from the secondary damage caused by inflammation [27], and the mechanical damage caused by the swelling itself, a boost of IV corticosteroids (as opposed to oral treatment) would be more efficient, as was found for optic neuritis in the ONTT [4]. Our results were disappointing in that IV corticosteroids for NAION improved neither the VA nor the VF of NAION patients compared to untreated patients.

Of further concern is the list of systemic side effects of corticosteroids and significance of diabetes instability, hypertensive crisis, weight gain and mood instability (although these conditions were not clearly shown in our small group) when dealing with a population that initially is at risk, even when on occasion the classic cause-effect relationship is not so obvious. Hence we believe that comprehensive treatment of NAION patients with systemic corticosteroids will be acceptable only when a large, prospective, randomized, multicenter study will prove the clear benefit of treatment, as was shown in the ONTT for optic neuritis.

Unfortunately, there are many limitations to our study. The major limitation is the retrospective nature of this study which does not allow deducing clear conclusions about corticosteroid treatment for NAION. Another major limitation is the relatively small group of patients. Furthermore, only patients with poor visual parameters, as well as those with low vision in the fellow eye were offered IV corticosteroid treatment which leads to a selection bias. Moreover, the control group included also patients with contraindications for steroid treatment, such as uncontrolled diabetes or hypertension, which can potentially worsen the final visual results. The power of our study is its relatively long follow-up and matched controls, as far as can be performed retrospectively.

## Conclusion

In summary, our data, with the major limitations stated above, does not suggest the superiority of IV steroid treatment over no treatment. As intravitreal corticosteroid treatment for NAION is still anecdotal [28,29], any steroid protocol for NAION patients should be used judicially. In our center, based on our experience, we chose to abandon the use of IV corticosteroids for NAION patients until a randomized control trial will prove the benefit of such treatment.

## Competing interests

The authors declare that they have no competing interests.

## Authors’ contributions

RHB conceived of the study. MK, YW, IBB and RHB participated in the design of the study and data collection. MK drafted the manuscript. AC performed the statistical analysis. All authors read and approved the final manuscript.

## Pre-publication history

The pre-publication history for this paper can be accessed here:

http://www.biomedcentral.com/1471-2415/14/62/prepub
